# Synaptic Ribbon Active Zones in Cone Photoreceptors Operate Independently from One Another

**DOI:** 10.3389/fncel.2017.00198

**Published:** 2017-07-11

**Authors:** Justin J. Grassmeyer, Wallace B. Thoreson

**Affiliations:** ^1^Department of Pharmacology and Experimental Neuroscience, University of Nebraska Medical Center Omaha, NE, United States; ^2^Truhlsen Eye Institute and Department of Ophthalmology and Visual Sciences, University of Nebraska Medical Center Omaha, NE, United States

**Keywords:** ribbon synapse, retina, exocytosis, calcium imaging, cone photoreceptor, active zone

## Abstract

Cone photoreceptors depolarize in darkness to release glutamate-laden synaptic vesicles. Essential to release is the synaptic ribbon, a structure that helps organize active zones by clustering vesicles near proteins that mediate exocytosis, including voltage-gated Ca^2+^ channels. Cone terminals have many ribbon-style active zones at which second-order neurons receive input. We asked whether there are functionally significant differences in local Ca^2+^ influx among ribbons in individual cones. We combined confocal Ca^2+^ imaging to measure Ca^2+^ influx at individual ribbons and patch clamp recordings to record whole-cell I_Ca_ in salamander cones. We found that the voltage for half-maximal activation (V_50_) of whole cell I_Ca_ in cones averaged −38.1 mV ± 3.05 mV (standard deviation [SD]), close to the cone membrane potential in darkness of ca. −40 mV. Ca^2+^ signals at individual ribbons varied in amplitude from one another and showed greater variability in V_50_ values than whole-cell I_Ca_, suggesting that Ca^2+^ signals can differ significantly among ribbons within cones. After accounting for potential sources of technical variability in measurements of Ca^2+^ signals and for contributions from cone-to-cone differences in I_Ca_, we found that the variability in V_50_ values for ribbon Ca^2+^ signals within individual cones showed a SD of 2.5 mV. Simulating local differences in Ca^2+^ channel activity at two ribbons by shifting the V_50_ value of I_Ca_ by ±2.5 mV (1 SD) about the mean suggests that when the membrane depolarizes to −40 mV, two ribbons could experience differences in Ca^2+^ influx of >45%. Further evidence that local Ca^2+^ changes at ribbons can be regulated independently was obtained in experiments showing that activation of inhibitory feedback from horizontal cells (HCs) to cones in paired recordings changed both amplitude and V_50_ of Ca^2+^ signals at individual ribbons. By varying the strength of synaptic output, differences in voltage dependence and amplitude of Ca^2+^ signals at individual ribbons shape the information transmitted from cones to downstream neurons in vision.

## Introduction

Photoreceptors release glutamate-laden vesicles at rates continuously regulated by graded, light-driven changes in membrane voltage (*V*_m_). To sustain this continuous and abundant output, the number of vesicles in a photoreceptor terminal exceeds that of a conventional hippocampal synapse by more than two orders of magnitude and photoreceptors possess specialized presynaptic structures called ribbons that organize and coordinate vesicular release (Sterling and Matthews, 2005; Schmitz, 2009). Cones can have up to 50 ribbon-style active zones that are typically separated from one another by 500 nm or more within the synaptic terminal (DeVries et al., 2006), while mammalian rods generally possess only a single ribbon (Carter-Dawson and LaVail, 1979; Sterling and Matthews, 2005). Synaptic vesicles are tethered to a ribbon by thin filaments but can move freely along the ribbon surface (Graydon et al., 2014). Beneath each ribbon sits a cluster of L-type Ca^2+^ channels and other presynaptic proteins that control the exocytosis of synaptic vesicles (Mercer and Thoreson, 2011; Lv et al., 2016; Maxeiner et al., 2016). Ribbon-localized Ca^2+^ channels mediate nearly all of the photoreceptor Ca^2+^ current (I_Ca_; Mansergh et al., 2005; Xu and Slaughter, 2005; Mercer and Thoreson, 2011). The opening of Ca^2+^ channels generates large increases in Ca^2+^ just beneath the ribbon, with the subsequent Ca^2+^ spread determined by both the magnitude of influx and intracellular buffering conditions (Choi et al., 2008; Van Hook and Thoreson, 2015). Vesicular release from cones, like many other CNS synapses (Brandt et al., 2005; Goutman and Glowatzki, 2007; Jarsky et al., 2010; Eggermann et al., 2011), is regulated by Ca^2+^ levels attained only within highly localized nanodomains adjacent to open Ca^2+^ channels (Bartoletti et al., 2011). Nanodomain control of release and the fact that ribbon-associated Ca^2+^ signals can remain spatially restricted from one another suggest that differences in Ca^2+^ dynamics among individual photoreceptor ribbons could diversify signals transmitted to postsynaptic neurons.

Ribbon-bearing cochlear inner hair cells exhibit significant differences in the amplitude and voltage dependence of ribbon-localized Ca^2+^ signals that are thought to contribute to diversity in firing rate, sound threshold, and dynamic range among second-order spiral ganglion neurons (Frank et al., 2009; Ohn et al., 2016). Significant differences in Ca^2+^ influx arising from local differences in the function or subunit composition of Ca^2+^ channels at different hippocampal synapses have also been observed (Éltes et al., 2017). Voltage dependence might also vary at different synapses as a consequence of local differences in the ionic environment and neuromodulatory influence. Changes of only a few millivolts in the midpoint activation voltage (V_50_) for I_Ca_, such as those caused by inhibitory feedback from horizontal cells (HCs; Verweij et al., 1996), can produce functionally significant changes in synaptic output from cones. In this study, we asked whether ribbons within individual cones show functionally significant differences in the voltage dependence of ribbon-associated Ca^2+^ channels, allowing them to operate independently from one another. Alternatively, all ribbons within an individual cone may exhibit the same voltage dependence and function more like a single distributed ribbon.

To assess the functional independence of Ca^2+^ responses at individual ribbon synapses in cones, we combined whole-cell patch clamp recordings of I_Ca_ with confocal imaging of individual ribbons in a vertical slice preparation of salamander retina. After accounting for cone-to-cone differences and potential technical sources of variability, we found significant intrinsic differences in the amplitude and voltage dependence of Ca^2+^ influx among ribbons within individual cones. In the absence of negative feedback from HCs, the intrinsic variability in V_50_ values among ribbons (standard deviation [SD] = 2.5 mV) is sufficient to produce differences in Ca^2+^ influx at individual ribbons of 45% or more at a cone’s resting membrane potential in darkness. We assessed the sensitivity of confocal Ca^2+^ measurements by manipulating extracellular pH to produce 3 mV shifts in voltage dependence. By directly manipulating HC membrane potential in paired whole-cell recordings with cones, we confirmed the ability of ribbons operate independently by showing that inhibitory feedback from an individual HC was capable of altering Ca^2+^ responses at some ribbons but not others in a cone terminal (Thoreson and Mangel, 2012). In contrast to the traditional view that the membrane potential in cones in darkness sits near the foot of the I_Ca_ activation curve (Barnes and Kelly, 2002), we found that the average V_50_ for whole-cell I_Ca_ and ribbon Ca^2+^ signals was near the dark resting membrane potential. This arrangement is optimal for signaling small changes in voltage produced by small changes in illumination (Sterling and Laughlin, 2015). Ribbon-to-ribbon differences in voltage dependence and amplitude of Ca^2+^ responses can produce ribbon-to-ribbon differences in synaptic gain and thus expand the transformations available to an individual cone for encoding light-evoked voltage responses into changes in synaptic release. This may improve the ability of a cone to signal luminance changes over a wide range of intensities.

## Materials and Methods

### Animal Care and Use

All experiments were performed using *ex vivo* vertical slices of retina from aquatic tiger salamanders (*Ambystoma tigrinum*; Charles Sullivan Co., Nashville, TN, USA) of both sexes (18–25 cm in length). We used cones from salamander retina for these studies because the ribbons in these cells are sufficiently far apart to be distinguished by optical imaging and because the synaptic terminal sits at the base of the cell body where it can be effectively voltage-clamped. All animal care and handling protocols were approved by the University of Nebraska Medical Center Institutional Animal Care and Use Committee. Euthanasia was conducted in accordance with AVMA Guidelines for the Euthanasia of Animals. Salamanders were kept on a 12-h dark/light cycle at 4–8°C.

### Slice Preparation

One to two hours after onset of the dark cycle, salamanders were euthanized by decapitation and rapidly pithed. The eyes were enucleated, the retina isolated, and vertical retinal slices (125 μm thickness) were prepared as described in detail elsewhere (Van Hook and Thoreson, 2013).

### Patch Clamp Electrophysiology

Throughout the experiments, the slice chamber was superfused at ~1 ml/min with chilled amphibian saline solution bubbled with 100% O_2_ (HEPES solution, standard conditions) or 95% O_2_/5% CO_2_ (${\text{HCO}}_{3}^{-}$ solution, when noted in text). The HEPES-buffered saline contained (in mM): 116 NaCl, 2.5 KCl, 1.8 CaCl_2_, 0.5 MgCl_2_, 5 glucose and 10 HEPES. The pH of this solution was adjusted to 7.8 (or 7.6 when noted in text) with NaOH. The bicarbonate-buffered solution contained (in mM): 101 NaCl, 2.5 KCl, 2.0 CaCl_2_, 0.5 MgCl_2_, 11 glucose and 22 NaHCO_3_. For both solutions, the osmolality was measured with a vapor pressure osmometer (Wescor) and if necessary adjusted to ~245 mOsm. Patch pipettes were pulled with a PP-830 or PC-10 vertical pipette puller (Narishige) from borosilicate glass pipettes (1.2 mm OD, 0.9 mm ID, with internal filament; World Precision Instruments) and had resistances of 9–15 MΩ. For some experiments, pipette shafts were coated with dental wax to reduce stray capacitance. The intracellular pipette solution contained (in mM): 50 CsGluconate, 40 CsGlutamate, 10 TEACl, 3.5 NaCl, 1 CaCl_2_, 1 MgCl_2_, 9.4 MgATP and 0.5 GTP-Na, 5 EGTA. After salts were dissolved, the pH was adjusted to 7.2 with CsOH (osmolality = 235–240 mOsm). All chemical reagents were from Sigma-Aldrich unless indicated otherwise.

Experiments were performed on an upright fixed-stage microscope (Nikon E600FN) under a water-immersion objective (60×, 1.0 NA). Cells were identified morphologically and recording electrodes were positioned with Huxley-Wall micromanipulators (Sutter Instruments). After obtaining a gigaohm seal, the patch was ruptured with gentle suction. Photoreceptor and HC recordings were conducted in voltage clamp and were performed using Alembic VE-2 (Alembic Instruments) and Axopatch 200B (Axon Instruments/Molecular Devices) amplifiers, respectively. Cone membrane currents from the Alembic were low-pass filtered at 3 kHz and HC currents from the Axopatch were filtered at 2 kHz. Some membrane currents were low-pass filtered at 200 Hz to facilitate data presentation. Signals were digitized with a Digidata 1322A (Axon Instruments, Molecular Devices) and acquired with pClamp 10 software (Molecular Devices). Series resistance was maximally compensated in every cone before recording using the Alembic amplifier, which allows stable compensation (Sherman et al., 1999).

Whole-cell I_Ca_ was measured using a ramp voltage protocol (−99 to +51 mV, 0.5 mV/ms) applied from a steady holding potential of −79 mV. Voltage-dependent and Ca^2+^-activated K^+^ currents in cones were blocked by TEA in the pipette solution and hyperpolarizing-activated cation currents (I_h_) were inhibited by Cs^+^ (Barnes and Hille, 1989). Ca^2+^-activated chloride currents were reduced by use of 5 mM EGTA but also activate slowly during the ramp protocol and are therefore only evident after the voltage ramp has moved above +20 mV (unpublished observations). For determination of V_50_ values, passive membrane resistance was subtracted from ramp currents using P/8 subtraction. Comparison of currents obtained in the presence of and absence of Cd^2+^ (0.1 mM) yielded the same current/voltage profiles for photoreceptor I_Ca_ (Stella and Thoreson, 2000). Consistent with earlier results (Stella and Thoreson, 2000), we also found that when we compared I_Ca_ evoked during the sequence of voltage steps used for Ca^2+^ measurements (described below) to I_Ca_ measured using the ramp voltage protocol (*n* = 6 cones), we observed the same voltage dependence with both measurements.

Most of the Ca^2+^ imaging experiments described in this study used 175 ms depolarizing steps applied from a steady holding potential of −79 mV (−39 to −19 mV, 5 mV increments) with 2 s between each step. We compared results obtained when the steps were applied in both forward and reverse order (forward: −39 to −19 mV, reverse: −19 to −39 mV). In trials where steps were applied in the forward order, ribbon Ca^2+^ signals yielded V_50_ values that were 2.06 ± 1.38 mV more positive than whole cell I_Ca_ measured in the same cones (*n* = 10 ribbons, *n* = 7 cones). In trials where steps were applied in the reverse order, ribbon Ca^2+^ signals yielded V_50_ values that were 2.17 ± 0.97 mV more negative than whole cell I_Ca_ (*n* = 10 ribbons, *n* = 7 cones). Values from the two different measurement sequences thus straddled the values for I_Ca_. Photoreceptor I_Ca_ in salamander rods shows slow and weak Ca^2+^-dependent inactivation (τ = 1.7 s; Corey et al., 1984; Rabl and Thoreson, 2002). To reduce the potential impact of Ca^2+^-dependent inactivation, we performed step series in the forward order. As addressed in the results, the more-positive V_50_ values obtained when steps are applied in the forward direction results at least in part from using the low affinity Ca^2+^ dye that does not readily detect small Ca^2+^ changes at weak voltages. For some of the later experiments on HC to cone feedback, we also used a staircase protocol in which we did not return to baseline (−79 mV) between test voltages to speed data acquisition during paired recordings. Voltage dependence measured with the two protocols did not differ noticeably.

Whole cell membrane resistance and capacitance in cones averaged 192 ± 88.9 MΩ and 80 ± 16.4 pF, respectively (mean ± SD, *n* = 47 cones). We excluded recordings if the current needed to voltage clamp a cone at −79 mV exceeded 250 pA or if the series resistance prior to compensation exceeded 60 MΩ. When multiple recordings within a cell were compared or averaged (e.g., when comparing ramp I_Ca_ with step-evoked Ca^2+^ signals), only trials with holding currents within 50 pA of one another were compared. When multiple stimulus trials were conducted within a cell, we waited at least 1 min between trials. We corrected membrane voltage values for a measured liquid junction potential by subtracting 9 mV.

### Confocal Ca^2+^ Imaging

**Figure 1:**
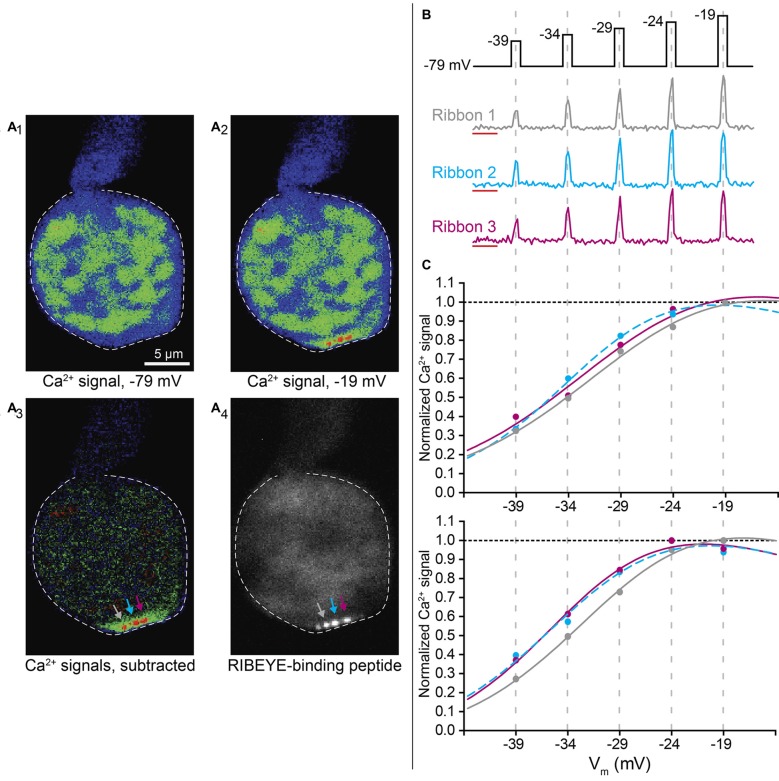
Depolarizing steps result in areas of localized Ca^2+^ influx at cone ribbons. **(A)** Example of a cone loaded with the low-affinity Ca^2+^ indicator OGB-5N and TAMRA-conjugated RIBEYE-binding peptide. A series of depolarizing steps was delivered to the cell to stimulate Ca^2+^ influx (from −79 mV to test potentials ranging from −39 mV to −19 mV in 5 mV increments, 175 ms apiece). Panel **(A_1_)** shows the pseudocolor fluorescence image (single 57 ms frame) of OGB-5N fluorescence in a cone prior to stimulation. Panel **(A_2_**) shows an image from the same cone during a depolarizing step to −19 mV. Panel **(A_3_)** shows the difference image obtained by subtracting the control image in **(A_1_)** from the test image in **(A_2_)**, revealing three distinct sites of Ca^2+^ influx in the terminal (arrows). In this cone, TAMRA-conjugated RIBEYE-binding peptide was also delivered by patch pipette, and the labeled ribbons observed at the same confocal plane are shown in panel **(A_4_)**. **(B)** The cone voltage stimulation protocol and Ca^2+^ responses of the three ribbons shown in Panel **(A)**. Traces show the fractional change in fluorescence over baseline (∆F/F). The region used to measure baseline fluorescence during the first 1.3 s is shown by the red lines. The graphs show ∆F/F measured in the three ribbons simultaneously during a single trial of the voltage stimulus protocol. **(C)** Data from two separate trials in this same cone showing peak ∆F/F values for each depolarizing stimulus plotted against test step potential after normalizing to the ribbon’s maximum ∆F/F within the trial. The voltage dependence was determined by fitting these data with a Boltzmann function adjusted for Ca^2+^ driving force (lines). Best fit V_50_ and slope factor values for trial 1: ribbon 1, −28.94 mV (95% confidence interval: −32.25 to −17.56 mV), 7.145; ribbon 2, −32.61 mV (−5.03 to −30.20), 5.616; ribbon 3, −29.91 mV (−42.25 to −17.562), 7.214. Best fit values for trial 2: ribbon 1, −30.46 mV (−33.75 to −27.17 mV), 6.240; ribbon 2, −33.61 mV (−40.36 to −26.86), 6.028; ribbon 3 −33.93 mV (−37.07 to −30.79), 5.621.

Confocal imaging was performed with Nikon Elements software using a laser confocal scanhead (Perkin Elmer Ultraview LCI) equipped with a cooled CCD camera (Orca ER) mounted on the Nikon E600FN. Excitation at 488 or 568 nm was delivered from an argon/krypton laser and emission was collected at 525 or 600 nm, respectively, by a cooled CCD camera (Hamamatsu OrcaER). Filters were controlled using a Sutter Lambda 10–2 filter wheel and controller. The objective was controlled using a E662 z-axis controller (Physik Instrumente). Cell-impermeant Ca^2+^ indicators Oregon Green 488 BAPTA-5N (OGB-5N, *K*_d_ = 20 μM, Thermo Fisher) or Oregon Green 488 BAPTA-6F (OGB-6F, *K*_d_ = 3 μM, Thermo Fisher) were added to the patch pipette solution at 400 μM. Images (57 ms/frame) were acquired during the voltage step protocol described above and analyzed using Nikon Elements 4.5 and Microsoft Excel software. Fluorescence values were measured as the mean pixel intensity within the region of interest (ROI). Baseline Ca^2+^ signal fluorescence was calculated by averaging signals during the first 1.3 s of the imaging trial when the photoreceptor was voltage clamped steadily at −79 mV. Fluorescence changes (∆F) evoked by depolarizing voltage steps were compared to baseline fluorescence (F) and the resultant ∆F/F ratios were normalized to the largest fluorescence change in each trial before using nonlinear regression to determine V_50_ values (see Figure 1). Reduction in ∆F/F amplitude induced by activation of HC negative feedback during paired recordings was quantified by comparing the maximum Ca^2+^ ∆F/F change in the absence of feedback to ∆F/F elicited by the same stimulus in the presence of feedback. Ribbons were defined as being subject to negative feedback if ∆F/F was reduced during HC depolarization in at least four out of five depolarizing steps (−39 to −19 mV) in two consecutive trials.

We measured the optical resolution of our system using fluorescent microbeads. The full width half maxima (FWHM; 2.355 × SD) of Gaussian functions fit to the fluorescence profiles in the x-y plane averaged 318 and 455 nm for 525 and 600 nm emission, respectively. To measure resolution in the z-axis, we acquired a z-stack and measured the fluorescence profile as a function of depth. The FWHM in the z-axis averaged 1.45 and 1.46 μm for 525 and 600 nm emission, respectively.

### Data Analysis

Activation profile data from Clampfit, Nikon Elements, and Microsoft Excel was imported to GraphPad Prism to perform nonlinear regression, for statistical analyses, and to generate figures. To calculate V_50_ values and construct I_Ca_ activation curves (G/G_max_), Ca^2+^ responses were fit with Boltzmann functions adjusted for Ca^2+^ driving force according to the equation

$$I\;=\;\left(V_{\text{rev}}-\;V_{\text{m}}\right)\;*\;\frac{I_{\max}}{1\;+\;e^{\frac{\left(V_{50}-V_{\text{m}}\right)}{k}}}$$

where *V*_rev_ = +41 mV and *k* is the slope factor of the voltage-dependent activation process. *V*_rev_ was the only constrained parameter. Distribution normality was assessed by D’Agostino-Pearson Test. Data is reported as mean ± SD unless indicated otherwise.

## Results

### Small Differences in the Activation of Ribbon Ca^2+^ Influx can be Detected

To visualize Ca^2+^ influx at individual ribbon sites, we used spinning disk confocal microscopy while simultaneously controlling the membrane voltage of individual cones by voltage clamp. We introduced a low-affinity Ca^2+^ indicator, Oregon Green BAPTA-5N (OGB-5N, *K*_d_ = 20 μM), into photoreceptors through the patch pipette and then evoked Ca^2+^ entry by delivering a series of depolarizing voltage steps. Ca^2+^ signals elicited in this way were typically constrained to hotspots near ribbons and only well-localized Ca^2+^ hotspots were analyzed (Zenisek et al., 2003; Choi et al., 2008; Frank et al., 2009; Mercer and Thoreson, 2011; Ohn et al., 2016). Figure 1A shows an example from one cone in which three Ca^2+^ hotspots were imaged simultaneously in the same confocal plane. Figure 1A_1_ shows an image obtained prior to depolarizing stimulation while panel **A**_2_ shows an image obtained during a depolarizing step to −19 mV. Subtracting the image in panel **A**_1_ from that in **A**_2_ yielded the difference image shown in **A**_3_, in which three Ca^2+^ hotspots are clearly visible (arrows). In this cell, we also labeled ribbons by introducing a RIBEYE-binding peptide conjugated to tetramethylrhodamine (TAMRA) through the patch pipette (Zenisek et al., 2004). After recording a series of depolarization-induced Ca^2+^ signals, we obtained a z-stack image to visualize ribbons in the same cone. The three Ca^2+^ hotspots shown in Figure 1A_3_ correspond to three distinct fluorescently-labeled ribbons at the plane used for Ca^2+^ imaging (Figure 1A_4_). A fourth ribbon that was less strongly labeled was also visible in the same plane but did not produce a discrete Ca^2+^ hotspot.

Figure 1B illustrates the stimulation protocol used in this and most other experiments by showing ribbon-localized Ca^2+^ responses evoked with a series of depolarizing test steps for the three ribbons in Figure 1A. At each of these ribbons, Ca^2+^-dependent fluorescence rose quickly during each depolarizing step and then returned quickly to baseline after the step. The peak fluorescence change (∆F/F) elicited at each stimulation step was normalized to the maximum ∆F/F signal within each trial. The change in ∆F/F as a function of voltage was then fit with a Boltzmann function modified for Ca^2+^ driving force. Each ribbon site was analyzed independently. Figure 1C shows results from two consecutive stimulation trials in the same cone. In both trials, ribbons 1 and 2 showed a similar difference in voltage dependence from one another, with their half maximal voltage of activation (V_50_) values differing by 3.67 and 3.15 mV in trials 1 and 2, respectively. In trial 1, the V_50_ value for ribbon 1 fell outside the 95% confidence interval for ribbon 2. The voltage dependence of ribbon 3 differed more between the two trials, with the difference in V_50_ between ribbons 1 and 3 increasing from 0.32 mV to 2.70 mV between trials 1 and 2. In trial 2, the V_50_ value for ribbon 1 fell outside the 95% confidence interval for ribbon 3 and vice versa. Observations from eight other cones also showed reproducible differences in V_50_ values between ribbons in the same cone suggesting that the voltage dependence of ribbon Ca^2+^ responses may genuinely differ from one another. In the experiments described below, we quantified these V_50_ differences after assessing various factors that might confound measurement accuracy.

### Evaluating Accuracy of Measuring Ribbon-Localized Ca^2+^ Responses

To measure Ca^2+^ changes at individual ribbons, we drew regions of interest (ROIs) around each ribbon-associated hotspot, tightly delineating regions with clear and distinct Ca^2+^ increases. In experiments where we also included a fluorescent RIBEYE-binding peptide in the patch pipette to label ribbons (e.g., Figure 1A_4_), ROIs for Ca^2+^ hotspots were defined by outlining the region of peptide fluorescence in the same confocal plane.

**Figure 2:**
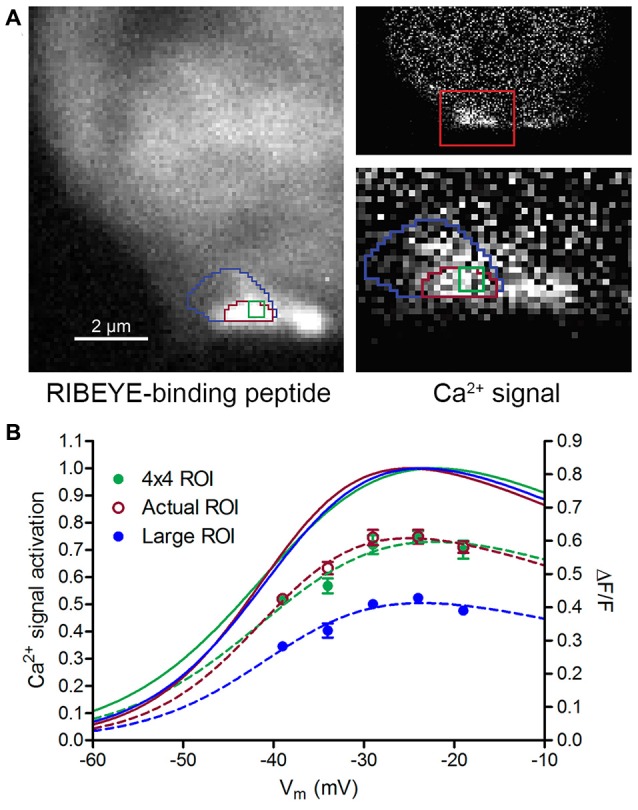
Impact of region of interest (ROI) size on Ca^2+^ signal measurements. **(A)** Three measurement ROIs superimposed on images of fluorescent RIBEYE-binding peptide (left) and the Ca^2+^ signal difference image (right) obtained by subtracting baseline OGB-5N fluorescence at −79 mV from a Ca^2+^ response at −19 mV. Higher magnification image (width = 5 μm) at the bottom right of panel **(A)** shows a close-up view of the ribbon-associated Ca^2+^ hotspot. The ROI with the magenta outline was drawn to correspond to the ribbon boundary as defined by RIBEYE-binding peptide fluorescence. The green ROI represents a 4 × 4 pixel (~0.4 × 0.4 μm) region at the center of ribbon and the large blue ROI encompasses areas surrounding the ribbon. **(B)** Ca^2+^ signal ∆F/F and voltage dependence of the three different-sized ROIs in Panel **(A)**. ∆F/F amplitude changes are plotted on the right axis. Boltzmann functions adjusted for driving force fit to ∆F/F values normalized to peak changes are plotted on the left axis. Although ∆F/F amplitudes were reduced considerably by use of the large ROI, the V_50_ values for the three different-sized ROIs were within 1.1 mV of one another (4 × 4, Actual, and Large ROI V_50_ values = −38.7, −39.8 and −39.0 mV, respectively). “Actual ROI” measurements were for the magenta ROI shown in panel **(A)**.

The size of an ROI can influence Ca^2+^ signal amplitude. To examine the impact of ROI area, we used RIBEYE-binding peptide fluorescence to define the ribbon boundary and then compared the amplitude of depolarization-evoked Ca^2+^ changes (∆F/F) as we varied ROI size around the ribbon (Figure 2A). As illustrated in Figure 2B, ∆F/F of Ca^2+^ responses decreased as ROI area was expanded to incorporate weakly responsive regions beyond the ribbon margins (blue border in Figure 2A). Using a very small ROI (4 × 4 pixels, ~0.4 × 0.4 μm, green border in Figure 2A) centered within the Ca^2+^ hotspot did not increase the amplitude of ∆F/F values beyond those measured with an ROI that delineated the entire ribbon (e.g., magenta border in Figure 2A). Use of such small ROIs placed in sub-regions did, however, increase measurement noise, so we integrated Ca^2+^ signals within the entire ribbon area to measure ribbon Ca^2+^ responses.

While changes in ROI size clearly affected ∆F/F amplitude, the normalized Ca^2+^ responses in Figure 2B show that the voltage dependence of Ca^2+^ signals was nearly identical for the three different-sized ROIs shown in Figure 2A. This is because voltage-dependent changes in Ca^2+^ fluorescence are strongly dominated by the signal at the center of the hotspot and so including additional weakly responsive regions had little impact. Similar comparisons performed in three other cones also showed that increasing ROI size diminished ∆F/F amplitude but did not appreciably alter V_50_ values for Ca^2+^ signals. Therefore, while differences in ROI size contributed to ∆F/F amplitude variability in our measurements, they did not appear to significantly alter V_50_ values.

**Figure 3:**
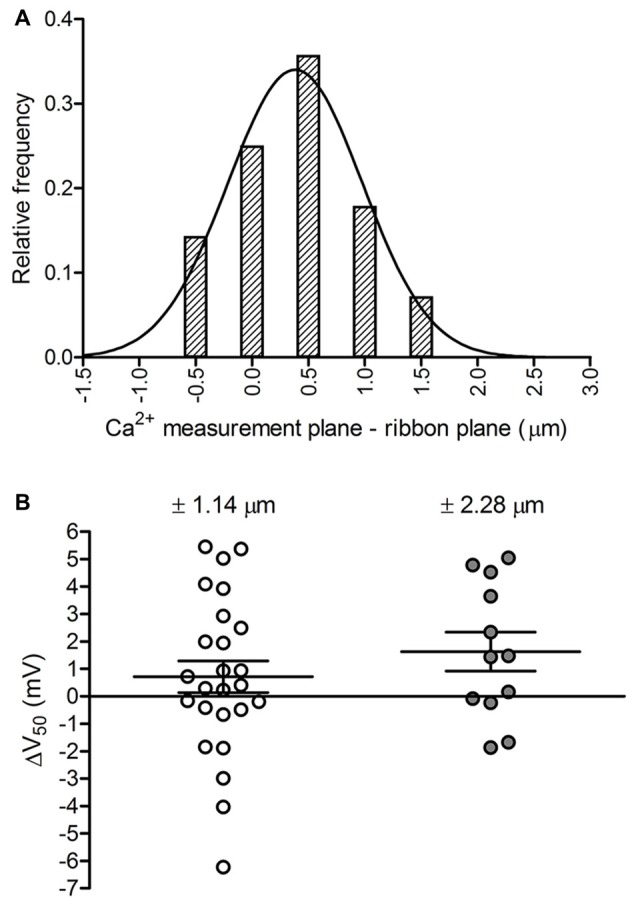
Assessing potential effects of focal plane placement on Ca^2+^ measurements. **(A)** Frequency distribution of the distance between the focal plane at which a ribbon’s Ca^2+^ signal was measured and the plane at which the peak fluorescence of TAMRA-conjugated RIBEYE-binding peptide was observed in a confocal z-stack acquired at the end of the recording (Gaussian mean = 0.38 μm, SD = 0.60 μm; fit *R*^2^ = 0.96;* n* = 28 ribbons in 11 cones). **(B)** Effect of focal plane position on V_50_ values. Moving the focal plane of Ca^2+^ measurement away from the ribbon plane ±1.14 μm shifted V_50_ values positively by +0.7 ± 2.9 mV (*p* = 0.23, paired *t*-test;* n* = 25 ribbons in 12 cones). Moving the plane ±2.28 μm shifted V_50_ values by +1.6 ± 2.5 mV (*p* = 0.04, paired *t*-test;* n* = 12 ribbons in 9 cones). Data are displayed as mean ± SEM.

We also examined the impact of confocal plane on measurements of ribbon Ca^2+^ signals. In experiments where we introduced fluorescent RIBEYE peptide through the patch pipette, we began Ca^2+^ measurements in the minutes before sufficient RIBEYE peptide had reached the synaptic terminal to produce strong ribbon labeling. Therefore, we typically selected the Ca^2+^ measurement plane by iterative positioning of the objective’s *z*-axis and application of depolarizing steps to locate discrete Ca^2+^ hotspots. To assess the accuracy of identifying ribbon-localized Ca^2+^ hotspots in this way, we compared the plane selected for Ca^2+^ measurements to the plane of the associated ribbon determined from a confocal z-stack of fluorescent RIBEYE peptide labeling (28 ribbons in 11 cones). Ca^2+^ measurements and z-stack images of RIBEYE fluorescence were typically acquired a few minutes apart from one another and small movements of the recording pipette or tissue during that time may have introduced additional displacements between the two measurements. Nonetheless, Ca^2+^ measurement planes and the brightest RIBEYE fluorescence planes differed by only 0.38 ± 0.60 μm (Figure 3A). When we examined the *z-axis* position of single fluorescent beads, we found that the beads imaged with 488 excitation/525 nm emission appeared to be 0.070 ± 0.066 μm (*p* = 0.009, paired *t*-test;* n* = 10 beads) above the same beads imaged with 568 nm excitation/600 nm emission. Taking these optical differences into account reduces the difference between measured planes to 0.31 ± 0.60 μm. This is less than the axial resolution of the 60×, 1.0 NA objective measured at 525 nm emission (1.45 μm). This shows good agreement between planes chosen for Ca^2+^ signal measurements and ribbon planes identified by RIBEYE peptide labeling.

These results show that Ca^2+^ signals were measured at or very near the actual ribbon plane, but we also considered how imperfect positioning of the focal plane of measurement might impact a ribbon’s calculated V_50_ value. To do so, we measured Ca^2+^ responses of a ribbon at one plane and then moved the objective up or down to make a second measurement. Fluorescent peptide was not included in these experiments and so we defined the ribbon plane *post hoc* as the plane showing the strongest Ca^2+^ response (largest ∆F/F). As illustrated in Figure 3B, movements of ±1.1 μm away from the plane of the ribbon caused a statistically insignificant V_50_ shift that averaged +0.7 ± 2.9 mV (*p* = 0.23) for 25 ribbons in 12 cones. A ±2.3 μm change in focal plane caused a larger, statistically significant positive shift in V_50_ of +1.6 ± 2.5 mV (*p* = 0.04). The ability of ±2.3 μm changes in focal plane to produce significant positive shifts in V_50_ values is likely a consequence of Ca^2+^ buffering and dye properties, such that greater Ca^2+^ influx is required to attain levels that can be accurately detected with the low-affinity Ca^2+^ dye.

Although large errors in focal plane placement can produce significant V_50_ shifts, these results suggest that iterative *z*-axis positioning between Ca^2+^ imaging trials to select the best focal plane resulted in ~95% of Ca^2+^ measurements being performed in a range spanning 1.2 μm about the ribbon plane. Trial-to-trial differences between V_50_ values of Ca^2+^ measurements made at different (±1.1 μm) focal planes did not differ significantly from trial-to-trial differences between V_50_ values obtained at the same focal plane (*p* = 0.44, *t*-test; *n* = 25 and 20 ribbons, respectively). This further indicates that focal plane selection had little or no impact on measurement variability. Taken together, these results show that Ca^2+^ signals were measured at focal planes that lay within the axial resolution limit to the true ribbon planes and that small differences among ribbon measurement focal planes did not introduce significant additional variability to V_50_ values.

Because both focal plane and ROI size can influence the amplitude of ∆F/F measurements in ribbons, we focused most of our analysis on ribbon-to-ribbon differences in voltage dependence. However, there were also genuine differences in Ca^2+^ influx magnitude among ribbons. For example, while peak ∆F/F values averaged 0.76 ± 0.77, one ribbon showed a peak ∆F/F value of 3.95, more than 4 SD above the average. This large Ca^2+^ increase was not due to use of a particularly small ROI nor can it be explained by focal plane positioning.

In experiments where we labeled ribbons with a fluorescent RIBEYE-binding peptide, we measured the length of the ribbon in the x-y plane by determining the distance between two points along the ribbon’s longest axis where fluorescence intensity had declined by 50% (full width at half maximum, FWHM). Measurements of ribbon length scale roughly with total ribbon size since the base of the ribbon is longer and varies more than the other two ribbon dimensions. Cone ribbons are 35–60 nm thick and extend <350 nm into the cytoplasm, but the length of a ribbon along its base can be well over 1 μm (Pierantoni and McCann, 1984; Pang et al., 2008). Fluorescently-labeled ribbons measured along their longest axis averaged 1.89 ± 0.87 μm (*n* = 26). The optical point spread function showed a FWHM of 0.45 μm. Therefore, after consideration of optical blurring, fluorescent measurements of ribbon length are consistent with ultrastructural measurements in light-adapted turtle cones, in which ribbon length averaged 1.2 ± 0.6 μm (Pierantoni and McCann, 1984). This supports the idea that individual Ca^2+^ hot spots generally arose from activity at individual ribbons.

**Figure 4:**
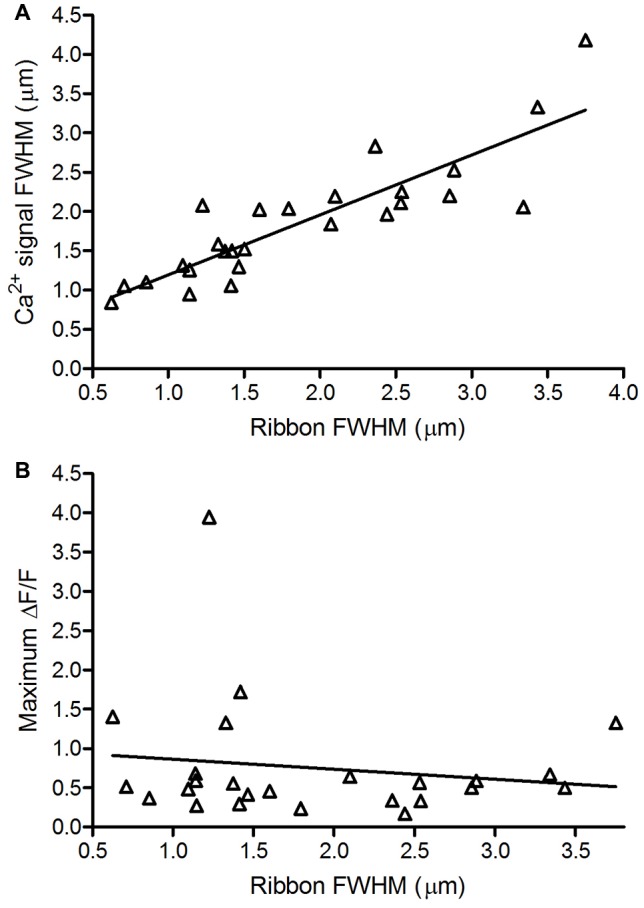
Ribbon size is correlated with Ca^2+^ domain size but not maximal Ca^2+^ response amplitude. **(A)** Spatial extent of Ca^2+^ signals (1.87 ± 0.77 μm, *n* = 26; measured as the FWHM of the peak depolarization-evoked OGB-5N fluorescence change) vs. size of the associated ribbon (*R*^2^ = 0.75; *p* < 0.0001, *F*-test for non-zero slope). **(B)** Maximum ∆F/F amplitude of Ca^2+^ signals vs. size of the associated ribbon. Maximum ∆F/F amplitude of Ca^2+^ signals averaged 0.76 ± 0.77 (*n* = 25). Ribbon size is represented by the full-width half maximum (FWHM) of the TAMRA-conjugated RIBEYE-binding peptide fluorescence measured along its longest axis in its brightest confocal plane (1.86 ± 0.90 μm, *n* = 25; *R*^2^ = 0.02; *p* = 0.48, *F*-test for non-zero slope).

The length of a ribbon along its base determines the number of vesicles that can be tethered at the plasma membrane and thus determines the size of the readily releasable pool of vesicles (Pang et al., 2008; Bartoletti et al., 2010). We predicted that the number of Ca^2+^ channels clustered beneath the base of each ribbon would also be correlated with the length of the ribbon. We therefore compared the length of each ribbon to the FWHM of the associated Ca^2+^ signal. As predicted, we found a strong linear correlation between ribbon size and the spatial extent of the associated Ca^2+^ signal (Figure 4A, *R*^2^ = 0.75).

In cochlear inner hair cells, the maximum amplitude of depolarization-evoked Ca^2+^ signals increased with increasing ribbon size (Frank et al., 2009; Ohn et al., 2016). We found no correlation between ribbon size and peak ∆F/F values attained in the associated Ca^2+^ hotspot (Figure 4B, *R*^2^ = 0.02) and the slope of the regression line did not differ significantly from zero (*F*-test, *p* = 0.48). The finding that Ca^2+^ signals are strongly correlated in spatial extent, but not peak amplitude, with ribbon size suggests that the number of Ca^2+^ channels generally scales with ribbon size.

**Figure 5:**
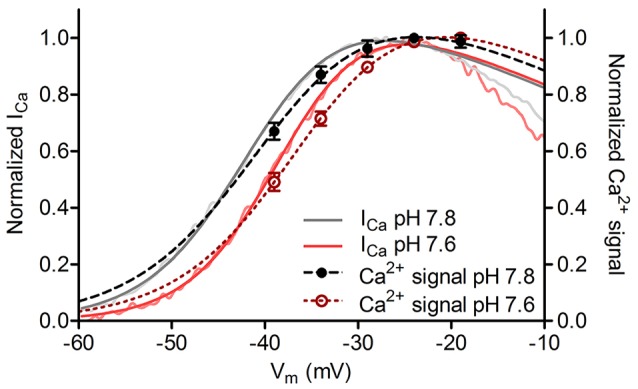
Small changes in voltage dependence of Ca^2+^ influx could be accurately detected. Cone I_Ca_ and ribbon Ca^2+^ signals plotted against test potential at extracellular pH values of 7.8 and 7.6. Changing the pH of extracellular saline from 7.8 (control condition) to 7.6 produced a positive shift in voltage dependence of ribbon Ca^2+^ responses (V_50_ shift = +3.3 mV, dashed lines) and whole cell I_Ca_ (V_50_ shift = +3.2 mV, solid lines; *p* = 0.03 for both comparisons, paired *t*-test;* n* = 8 ribbons in 7 cones). Normalized I_Ca_ traces (pink and light gray) were low-pass filtered at 200 Hz to facilitate visual comparison.

The voltage-dependent activation of L-type I_Ca_ can be altered changes in extracellular pH (Iijima et al., 1986; Krafte and Kass, 1988; Barnes et al., 1993). To assess our ability to accurately measure small changes in V_50_ from ribbon-associated changes in Ca^2+^, we altered the pH of the extracellular saline from 7.8 (control) to 7.6 to induce small changes in voltage dependence of I_Ca_ activation. Consistent with earlier measurements of the effect of pH on salamander cone I_Ca_ (Barnes and Bui, 1991; Barnes et al., 1993), lowering pH by 0.2 units shifted V_50_ values of I_Ca_ in cones by +3.2 mV (Figure 5, *p* = 0.03). This 0.2 unit pH change caused a readily detectable and significant shift in the V_50_ of Ca^2+^ signals measured optically at individual ribbons in the same cones that averaged +3.3 mV (Figure 5, *p* = 0.03). These small, reproducible changes in V_50_ induced by altering extracellular pH confirmed that small differences in Ca^2+^ signal voltage dependence could be reliably detected at individual ribbons in our experiments.

### Cone-to-Cone I_Ca_ V_50_ Variability

**Figure 6:**
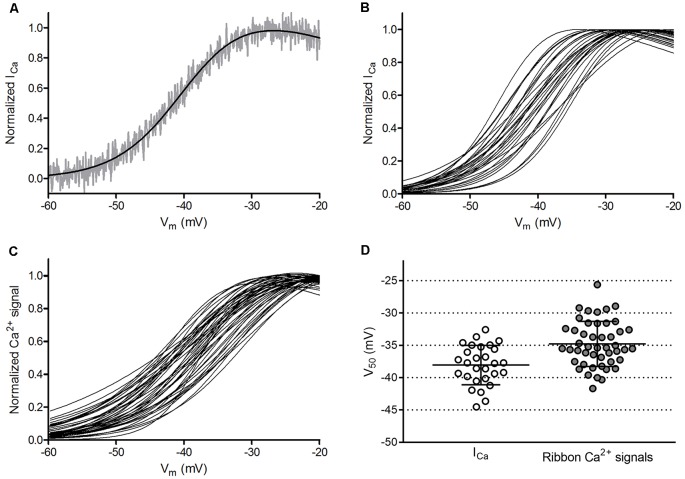
Ribbon-to-ribbon Ca^2+^ activation is more variable than cone-to-cone I_Ca_ activation. **(A)** I_Ca_ in one cone with R_ser_ completely compensated. I_Ca_ was normalized to its peak value and plotted against the cone holding potential during the voltage ramp protocol (gray trace). A Boltzmann function adjusted for driving force was fit to these data (black line, V_50_ = −39.3, slope factor = 4.88). Inward currents are plotted upward to compare more easily with Ca^2+^ signal measurements. For this illustration, currents were digitally corrected for the passive membrane resistance measured in the range from −85 mV to −70 mV. **(B)** Overlaid Boltzmann function fits to normalized I_Ca_ from the 28 cones in which ribbon Ca^2+^ signals in Panel **(C)** were measured. **(C)** Overlaid Boltzmann function fits to ribbon-associated Ca^2+^ signals of 47 ribbons in the 28 cones shown in Panel **(B)**. Ribbons were analyzed as illustrated in Figure 1. **(D)** Distribution of V_50_ values calculated from Boltzmann function fits to I_Ca_ (average = −38.1 ± 3.0 mV) and optical ribbon Ca^2+^ measurements made with OGB-5N (average = −34.8 ± 3.5 mV). Bars show the mean ± SD.

Cone-to-cone differences in the voltage dependence of I_Ca_ activation could be a source of post-synaptic variability in bipolar cells as well as a source of variability in V_50_ values when comparing ribbons in different cones. We therefore examined the variability of V_50_ values calculated from cone I_Ca_ activation curves measured electrophysiologically. Technical variability in measurements of I_Ca_ in different cones could arise from differences in series resistance (R_ser_) during whole-cell patch clamp recordings. To minimize this, we employed an amplifier (Alembic VE-2, Alembic Instruments) with circuitry that allows complete and stable compensation of R_ser_ (Sherman et al., 1999). We measured cone I_Ca_ using a ramp voltage protocol (−99 to +51 mV, 0.5 mV/ms) with P/8 subtraction of passive membrane properties and then fit the normalized I_Ca_ curve with a Boltzmann function adjusted for driving force. An example of I_Ca_ analyzed in this way is shown in Figure 6A, while Figure 6B shows overlaid Boltzmann fits of I_Ca_ from 21 cones. Even after compensating for R_ser_, we found modest differences in the voltage dependence of I_Ca_ from cone to cone, with an average V_50_ of −38.1 ± 3.05 mV (variance = 9.28 mV^2^, *n* = 28 cones) and slope factor of 4.90 ± 1.34. The peak amplitude also differed among cones, averaging 163.6 ± 41.6 pA (*n* = 28).

We analyzed trial-to-trial variability of V_50_ values obtained from repeated I_Ca_ measurements in the same cone, which produced an average variance of 1.0 mV^2^ (SD = 1.0 mV). The Alembic amplifier circuitry allows near complete compensation for R_ser_, but the Clampex membrane test evaluator suggested a small residual R_ser_ averaging 2.1 ± 0.58 MΩ (*n* = 36). In 14 of the 28 cones used for ribbon analysis, we calculated the voltage error that would be introduced by R_ser_ after accounting for both the steady holding current and the magnitude of the inward I_Ca_ at its activation midpoint. The voltage error in these recordings caused by residual, uncompensated R_ser_ averaged 0.54 ± 0.19 mV, indicating that the true V_50_ for the cones in our analysis is slightly more negative than the value reported above. Additionally, this analysis shows that variability in residual R_ser_ among cones had a negligible impact on variance (0.04 mV^2^) in I_Ca_ V_50_ values among cones.

Assuming a cone should have an unchanging I_Ca_ voltage dependence, the genuine biological variability for I_Ca_ V_50_ among cones would have a SD of 2.9 mV (variance = 8.24 mV^2^ after subtracting trial-to-trial and R_ser_ variance). Our recordings were largely performed in large single cones but also included some small single cones and double cones (Mariani, 1986; Sherry et al., 1998). V_50_ values for I_Ca_ showed a single Gaussian distribution (Figure 6), suggesting considerable overlap of voltage dependence among subtypes. Furthermore, in a separate set of experiments using the same ramp protocol to measure I_Ca_, we found no significant differences in V_50_ values among these three cone subtypes (*p* = 0.56, analyses of variance (ANOVA); *n* = 5 small single cones, 6 large single cones, 6 principal members of double cones). The traditional view is that the membrane potential for cones in darkness (−40 mV) rests near the foot of the activation curve for I_Ca_ (Barnes and Kelly, 2002), but our results indicate that I_Ca_ would attain approximately half-maximal activation in darkness. As discussed later, small cone-to-cone differences in resting membrane potential and I_Ca_ V_50_ values can produce functionally significant differences in Ca^2+^ influx and synaptic output.

### Ca^2+^ Signals at Cone Ribbon Sites Exhibited Small Differences in Voltage Activation

Cone synaptic terminals contain a dozen or more synaptic ribbons that are each potentially capable of providing distinct output channels to second-order horizontal and bipolar cells. To determine whether these channels all transmit signals with equal voltage dependence, we examined the Ca^2+^ activation profiles of many cone ribbon sites, including multiple ribbons from some cones. Figure 6C shows an overlay of the Ca^2+^ activation curves from 47 ribbons in the 28 cones shown in Figure 6B. The V_50_ values for cone ribbon Ca^2+^ signals averaged −34.8 ± 3.49 mV. Slope factors for the Boltzmann fit averaged 6.76 ± 1.73. This differed significantly (*p* < 0.0001) from slope values for I_Ca_ due to the non-linear binding properties of the Ca^2+^ dye. The distributions of V_50_ values calculated for cone I_Ca_ (Figure 6B) and ribbon Ca^2+^ signals within these cones (Figure 6C) are both plotted in Figure 6D, and both exhibited a normal distribution (*p* = 0.71 and 0.57, respectively, D’Agostino and Pearson omnibus normality test). Even though multiple ribbons were often measured in a single cone, V_50_ values of ribbon Ca^2+^ signals showed slightly greater variability than V_50_ values of cone I_Ca_. Thus, the voltage dependence of ribbon Ca^2+^ signals was more variable than would be expected if all ribbons within a cone operated identically.

The V_50_ values calculated from ribbon Ca^2+^ influx in cones were 3.3 mV more positive than V_50_ values determined from I_Ca_ (Figure 6D). We hypothesized that this rightward shift in activation was due to the low sensitivity of the low affinity Ca^2+^ indicator (OGB-5N, *K*_d_ = 20 μM), such that small Ca^2+^ influxes evoked by weak depolarization lie below the dye’s linear response range. To test this idea, we repeated our experiments using a slightly higher-affinity Ca^2+^ indicator, OGB-6F (*K*_d_ = 3 μM), and expanded the test step series to include weaker steps to −49 and −44 mV. In support of our hypothesis, the difference in V_50_ between Ca^2+^ signals and I_Ca_ was reduced to 2.3 mV when using OGB-6F (*n* = 25 ribbons in 11 cones). However, Ca^2+^ hotspots were less tightly constrained with this higher-affinity dye, so we used OGB-5N for experiments examining ribbon-to-ribbon differences in Ca^2+^ signals.

### Estimating Genuine Ribbon-to-Ribbon Variability

To analyze how much ribbons truly differ in voltage dependence from one another, we evaluated sources of technical and biological variability in our measurements. Variability in V_50_ values for ribbon-associated Ca^2+^ signals (SD = 3.49 mV, variance = 12.18 mV^2^, see Figure 6) could potentially arise from four sources: (1) technical variability in electrophysiological control of cone I_Ca_; (2) biological cone-to-cone variability in I_Ca_ activation; (3) technical variability in optical measurements of Ca^2+^ signals; and (4) biological ribbon-to-ribbon variability in ribbon-associated Ca^2+^ signals. The combined impact of sources 1 and 2 (technical and biological variability of I_Ca_ among cones) was captured by measurements of variability among whole cell I_Ca_ in different cones (V_50_ SD = 3.05 mV, variance = 9.28 mV^2^, *n* = 28 cones, see Figure 6D).

**Figure 7:**
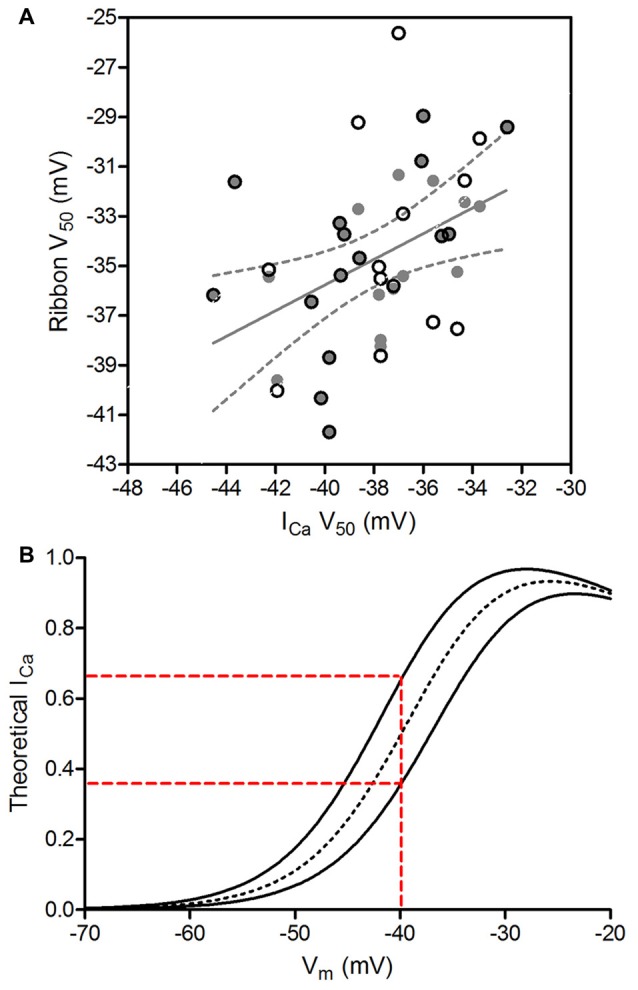
Genuine ribbon-to-ribbon variability can be functionally relevant. **(A)** V_50_ values of ribbon Ca^2+^ signals were not strongly correlated with V_50_ values of whole cell I_Ca_ from the cones in which they were measured. The strongest correlation was obtained by using one data point per cone and selecting the V_50_ values for ribbon Ca^2+^ signals that were most similar to V_50_ for I_Ca_ in the same cone (gray circles). The graph shows the linear regression fit to this subset of data along with the 95% confidence interval (*R*^2^ = 0.228, slope = 0.517 ± 0.187 [SEM]; *p* = 0.01, *F*-test for non-zero slope). Open circles show the single ribbons from each cone with V_50_ values that were least similar to V_50_ for I_Ca_ in the same cone. **(B)** Ribbons can exhibit functionally relevant differences in Ca^2+^ influx. The dotted black line simulates a ribbon with the average I_Ca_ parameters from our analysis (V_50_ = −38.1 mV, slope = 4.9, I_max_ = 0.0251). The two solid lines show curves differing in V_50_ by ±1 SD (V_50_ = −40.6 and −35.6 mV). The more positively-activating ribbon would experience >45% less Ca^2+^ influx than the more negatively-activating ribbon at a cone membrane potential of −40 mV (red dashed lines). Alternatively, the more negatively-activating ribbon would experience >80% more influx than the more positively-activating ribbon at −40 mV.

We assessed the amount of variance in Ca^2+^ signals among ribbons that can be explained by the variance in whole cell I_Ca_ among cones by examining the correlation between V_50_ values of cone I_Ca_ and V_50_ values of the ribbons measured in the same cones. We expected to find that much of the ribbon-to-ribbon variability would be explained by cone-to-cone variability in whole cell I_Ca_. However, we instead found that V_50_ values for ribbon Ca^2+^ signals were only weakly correlated with I_Ca_ V_50_ values from the cones in which they were measured (*R*^2^ = 0.105, slope = 0.39 ± 0.17 [SEM]; *p* = 0.026). To avoid weighting some cells more heavily than others, we repeated this comparison after choosing a single ribbon from each cell. Selecting a single ribbon from each cone to minimize the difference between ribbon V_50_ and I_Ca_ V_50_ (and thus maximize the correlation) improved the correlation to *R*^2^ = 0.228 (gray circles in Figure 7A; slope = 0.52 ± 0.19 [SEM]; *p* = 0.01). Selecting the one ribbon from each cone that maximized the difference between ribbon V_50_ and I_Ca_ V_50_ also slightly improved the correlation (open circles in Figure 7A; *R*^2^ = 0.164, slope = 0.52 ± 0.23 [SEM]; *p* = 0.03). These coefficients of determination indicate that 16.4%–22.8% of ribbon-to-ribbon variance can be attributed to variance in I_Ca_ among cones. Thus, while the amount of variance among V_50_ values for ribbon Ca^2+^ measurements was not significantly greater than the variance among V_50_ values for I_Ca_ (*F*-test, *p* = 0.46), the sources of variance in the two samples were largely independent.

We also assessed technical variability in optical measurements of Ca^2+^ signals (source 3). For each ribbon, we fit the voltage-dependent ∆F/F profiles with a Boltzmann function adjusted for driving force. We acquired up to five replicate data points for each depolarizing step in some experiments, and when such replicates were available, we fit the entire sample as one. The standard errors for V_50_ values obtained by nonlinear regression fits to ribbon Ca^2+^ signals provide a way to quantify uncertainties in V_50_ contributed by optical Ca^2+^ measurement variability. From the standard errors of Boltzmann fits to Ca^2+^ imaging data, we found that measurement uncertainty contributed an average variance of 3.61 mV^2^ (95% confidence interval: 2.35–4.87 mV^2^).

The genuine biological variability among ribbons was then determined by subtracting contributions from sources 1, 2 and 3 from the overall ribbon-to-ribbon variability. We found that 16.4–22.8% of the overall ribbon-to-ribbon variance arises from cone-to-cone variance in I_Ca_ (sources 1 and 2). Subtracting this contribution of cone-to-cone variance reduces ribbon-to-ribbon variance from 12.18 mV^2^ to 9.50–10.23 mV^2^. Subtracting the additional variance introduced by uncertainties in fitting Ca^2+^ responses (source 3: 3.61 mV^2^) leaves an estimated genuine biological ribbon-to-ribbon variance of 5.89–6.62 mV^2^ (SD = 2.4–2.6 mV). Employing this same analysis with data obtained from experiments with the Ca^2+^ dye OGB-6F yielded a similar ribbon-to-ribbon variance (5.96–6.48 mV^2^, SD = 2.4–2.5 mV). This range of variability is consistent with the example in Figure 1 showing differences in V_50_ averaging 3.3 mV between two ribbons in the same cone and indicates that the V_50_ values in a population of ribbons from a single cone are likely to span a range of at least 5.0 mV (±1 SD).

While a 5 mV range of V_50_ values may seem modest, an activation difference of this magnitude can alter Ca^2+^ influx by more than 45% when the cone is at a resting membrane potential in darkness of −40 mV. This is illustrated by the theoretical Ca^2+^ response curves shown in Figure 7B. We simulated the voltage dependence of I_Ca_ in cones using the average best-fit parameters of the I_Ca_ curves shown in Figure 6B (dashed line in Figure 7B), as well as curves with V_50_ values varying by ±1 SD from the mean (SD = 2.5 mV, solid lines in Figure 7B). At a membrane potential of −40 mV, a ribbon exhibiting the more positive Ca^2+^ activation function (V_50_ = −35.6 mV) that is 1 SD above the mean would experience 45% less Ca^2+^ influx than a ribbon exhibiting a more negative activation function (V_50_ = −40.6 mV) that is 1 SD below the mean. Extending this analysis to consider V_50_ values ±2 SD away from the mean indicated a difference in Ca^2+^ influx of 70% or more. Nearly identical differences in Ca^2+^ influx were obtained using average parameters of the curves fit to optical ribbon Ca^2+^ measurements (i.e., Figure 6C). In addition to altering the Ca^2+^ influx at a given membrane potential, changes in V_50_ will also alter the slope of the relationship between voltage and Ca^2+^ influx, thereby altering synaptic gain. At any given membrane potential, the slope of this relationship will differ for ribbons exhibiting different V_50_ values, producing ribbon-to-ribbon differences in synaptic gain.

### Horizontal Cell Feedback Modulates Ca^2+^ Influx at Individual Ribbons Within Cones

Inhibitory feedback from HCs regulates synaptic transmission from cones, contributing to the formation of center-surround receptive fields and color opponency in downstream retinal neurons (Thoreson and Mangel, 2012). Hyperpolarizing a HC, as occurs in light, reduces the peak amplitude of cone I_Ca_ and shifts its activation positively by a few millivolts (Verweij et al., 1996). In the experiments described above, we measured intrinsic differences among ribbons while blocking inhibitory synaptic feedback from HCs to cones by using a superfusate lacking bicarbonate and strongly buffered with 10 mM HEPES (Hirasawa and Kaneko, 2003). As a further test of the ability of cone ribbons to be regulated independently, we measured Ca^2+^ changes at individual ribbons while manipulating HC feedback during paired whole cell recordings between cones and postsynaptic HCs. For these experiments, we superfused the retinal slices with a bicarbonate-buffered medium that permits feedback (Warren et al., 2016a).

**Figure 8:**
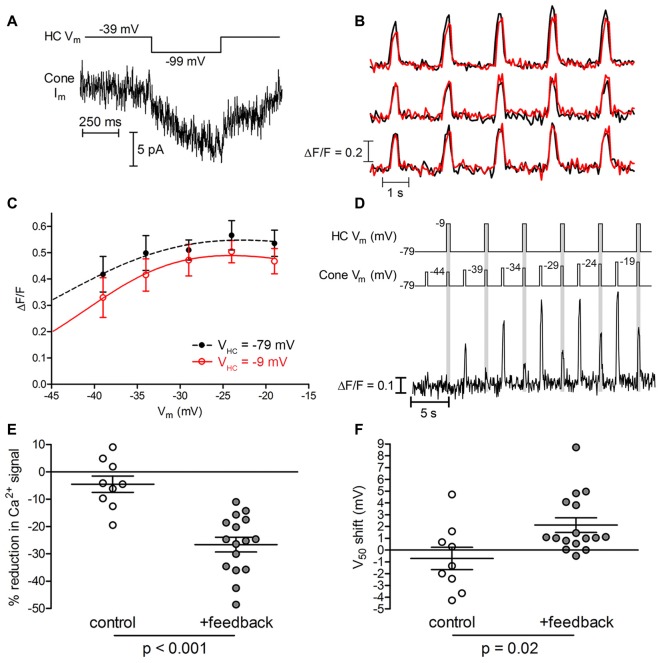
Negative feedback from horizontal cells (HCs) modulates cone ribbons independently. **(A)** HC-induced feedback current for the cone with ribbon Ca^2+^ responses illustrated in panels **(B,C)**. HC-to-cone feedback was confirmed electrophysiologically by holding the cone at −39 mV for 2 s to partially activate I_Ca_ and then testing whether hyperpolarizing the HC with a step from −39 mV to −99 mV to relieve inhibitory feedback stimulated an inward current arising from an increase in cone I_Ca_ (Warren et al., 2016b). **(B)** Ca^2+^ responses (∆F/F) measured simultaneously from three ribbons in a cone. Black traces show control responses obtained when the HC was voltage clamped at −79 mV. Red traces show responses obtained while activating inhibitory feedback to the cone by depolarizing the HC to −9 mV. In this cone, only one of the three ribbons (top set of traces) showed a reduction in the amplitude of Ca^2+^ influx when negative feedback was activated by depolarizing the HC. **(C)** Average amplitude (∆F/F) of Ca^2+^ signals plotted against cone membrane potential for the ribbon showing a feedback effect in panel **(B)**. When negative feedback was activated by depolarizing the HC to −9 mV, ribbon Ca^2+^ influx amplitude was decreased and the V_50_ shifted from −39.7 mV to −38.7 mV. **(D)** Alternative stimulus protocol for testing HC-mediated feedback effects. This example shows an exemplar ribbon’s Ca^2+^ responses during a sequence of depolarizing steps in the absence (white epochs) or presence (gray epochs) of inhibitory feedback from a simultaneously voltage-clamped HC. Test potentials delivered to the cone are indicated as Cone V_m_. A second sequence was subsequently performed in this pair but with HC depolarization applied first rather than second for each test step pair delivered to the cone. Measurements from the two trials were averaged to calculate the feedback-mediated amplitude reduction and V_50_ shift of Ca^2+^ responses at this ribbon. **(E)** Reduction in peak ∆F/F amplitude by HC negative feedback (feedback trials: −26.6 ± 10.6%; *p* < 0.001, paired *t*-test;* n* = 16 ribbons in 14 cones; control trials: −3.7 ± 12.6%; *p* = 0.4, paired *t*-test;* n* = 9 ribbons in 5 cones; *p* < 0.001 between conditions, *t*-test). **(F)** Magnitude of V_50_ shift in ribbon Ca^2+^ signals induced by depolarizing HCs from −79 mV to −9 mV (feedback trials: +2.1 ± 2.5 mV; *p* < 0.001, paired *t*-test;* n* = 16 ribbons in 14 cones; control trials: −0.7 ± 2.8 mV; *p* = 0.5, paired *t*-test;* n* = 9 ribbons in 5 cones; *p* = 0.02 between conditions, Mann Whitney test). Data are displayed as mean ± SEM in panels **(E,F)**.

We compared ribbon Ca^2+^ responses when a postsynaptic HC was voltage clamped at either −9 or −79 mV (i.e., with active or inactive inhibitory feedback, respectively). Before imaging, we first confirmed electrophysiologically that HC-to-cone feedback was present by holding the cone at −39 mV for 2 s to activate I_Ca_ and then hyperpolarizing the HC with a step from −39 mv to −99 mV to relieve inhibitory feedback. When feedback is present, hyperpolarizing the HC relieves feedback inhibition of cone I_Ca_ and stimulates an inward current (Figure 8A; Warren et al., 2016b). Figure 8B shows Ca^2+^ responses measured simultaneously at three different ribbons in one cone, with and without negative feedback from the HC (red and black traces, respectively). In this example, depolarizing the HC to activate inhibitory feedback reduced the amplitude of Ca^2+^ influx at one ribbon (top set of traces) but not at the other two. Presumably, dendrites from the voltage-clamped HC did not contact the two non-responsive ribbons. The Ca^2+^ changes at the ribbon subject to HC feedback are plotted in Figure 8C and show that depolarization of the HC reduced the magnitude of Ca^2+^ influx at every cone test potential. By fitting Boltzmann functions to these activation profiles, we also found that the V_50_ shifted positively by 1.0 mV when negative feedback was activated by depolarizing the HC. In this example, we held the HC at either −79 or −9 mV while testing cone responses over the entire voltage range (six replicate trials for each HC condition). Averaging multiple trials in this way reduced trial-to-trial changes in Ca^2+^ response amplitude.

In another approach to minimize trial-to-trial differences, we applied the same voltage step to some cones twice in succession, changing the HC holding potential from −79 mV to −9 mV (or vice versa) between the two steps (Figure 8D). In the example shown in Figure 8D, activating negative feedback by holding the HC at −9 mV strongly inhibited Ca^2+^ responses at this ribbon compared to responses evoked by the preceding test step applied when the HC was held at −79 mV. Strong inhibition of cone Ca^2+^ responses by inhibitory feedback from the HC in this example was evident despite the fact that in separate control trials conducted without a paired HC, the second in a pair of depolarizing test steps applied to the cone sometimes evoked a larger response than the first. To quantify feedback-evoked changes in amplitude and voltage dependence, we eliminated possible order effects of this protocol by averaging two sequences in which we swapped the order in which we applied the HC voltage changes. In one sequence, the first cone test step was applied while feedback was activated by holding the HC at −9 mV and the second cone test step was applied while holding the HC at −79 mV. In the other sequence, we reversed the order of HC test potentials to activate feedback during the second of each pair of cone test steps (e.g., Figure 8D).

Not every cone ribbon showed feedback-induced changes in Ca^2+^ signals. We saw feedback-evoked modulation at 16 out of 25 ribbons (64%) in 14 cone-HC pairs in which the presence of feedback was confirmed electrophysiologically (e.g., Figure 8A). In four of these cone-HC pairs, we saw changes at one ribbon but not another in the same cone. For ribbons that exhibited a feedback effect, inducing negative feedback by depolarizing the HC from −79 mV to −9 mV caused a significant reduction in peak Ca^2+^ signal ∆F/F amplitude at the 16 responsive ribbons (−26.6 ± 10.6%, *p* < 0.001). In identical control trials conducted without simultaneously voltage-clamped HCs, we saw no significant change in Ca^2+^ signal amplitude (*p* = 0.4); the difference between feedback and control conditions was significant (Figure 8E, *p* < 0.001). Depolarizing HCs from −79 mV to −9 mV also caused a significant positive shift in V_50_ (+2.1 ± 2.5 mV, *p* < 0.001) at ribbons sensitive to feedback, which differed significantly from control trials without a voltage-clamped HC (Figure 8F, *p* = 0.02). The ability of HCs to regulate Ca^2+^ signals at some ribbons but not others within a single cone provides further evidence that ribbons can operate in a functionally independent manner. Modulation by HC feedback could increase or diminish intrinsic differences in ribbon Ca^2+^ responses seen in the absence of feedback.

## Discussion

### Differences in Voltage Dependence and Amplitude of Ribbon Ca^2+^ Signals

Our experiments reveal significant ribbon-to-ribbon differences in the voltage dependence of ribbon-associated Ca^2+^ signals. After accounting for technical variability in Ca^2+^ signal measurements and contributions from cone-to-cone variability in I_Ca_, we found intrinsic variability (i.e., in the absence of inhibitory feedback from HCs) of V_50_ values among ribbon-localized Ca^2+^ signals with a SD of ~2.5 mV. Ribbon V_50_ values were normally distributed, suggesting that 68% (±1 SD) of ribbons in a cone exhibit V_50_ values spanning a range of 5 mV. We found a similar degree of ribbon-to-ribbon variability using the higher affinity Ca^2+^ dye OGB-6F. Consistent with this range of V_50_ values, we also observed reproducible V_50_ differences of up to 3.5 mV between neighboring ribbons in the same cone (e.g., Figure 1). Measurements of ribbon Ca^2+^ signals at two different pH values (7.6 and 7.8) confirmed that we could accurately detect 3 mV changes in voltage dependence (Figure 5). Functional independence of ribbons was also demonstrated by manipulating HC membrane potential to alter the strength of HC to cone feedback and thereby produce reversible shifts in the V_50_ and amplitude of Ca^2+^ responses at individual cone ribbons. Small variations in positioning the focal plane of measurement or the size of ROIs did not contribute significantly to V_50_ variability. Although seemingly modest, differences in activation voltage of a few millivolts can have a significant impact on Ca^2+^ influx. A typical cone dark resting membrane potential of −40 mV places I_Ca_ quite close to its activation midpoint. A difference in V_50_ values of 5 mV about that mean (±1 SD) would produce a difference in Ca^2+^ influx of more than 45%. Variation in the voltage dependence of whole-cell I_Ca_ would also produce cone-to-cone differences in Ca^2+^ influx. Because release from cones is linearly related to I_Ca_ (Jackman et al., 2009; Duncan et al., 2010; Bartoletti et al., 2011), ribbon-to-ribbon differences in Ca^2+^ influx would be expected to produce differences in release exceeding 45% at the dark resting potential.

We first assessed intrinsic differences in V_50_ variability among cone ribbons under conditions where inhibitory feedback from HCs to cones was blocked by use of the pH buffer HEPES (Hirasawa and Kaneko, 2003). In recordings where we allowed feedback to be active, directly depolarizing a voltage-clamped HC to stimulate inhibitory feedback in a simultaneously voltage-clamped cone shifted V_50_ of Ca^2+^ signals at cone ribbons by an average of +2.1 mV and reduced the amplitude of ribbon-associated Ca^2+^ responses by an average of 27%. HC feedback could therefore increase or diminish intrinsic differences in Ca^2+^ responses between individual ribbons in the same cone. Effects of feedback evoked by depolarizing a single HC were not exerted at every ribbon, but were instead restricted to a subset of ribbons within presynaptic cones.

We focused our study on differences in voltage dependence among ribbon-associated Ca^2+^ signals rather than amplitude. One major reason for this was that response amplitude measurements were more sensitive than V_50_ measurements to differences in placement of the focal plane or delineation of the ROI (Figures 2, 3). However, some of the differences in response amplitude observed between ribbons were far too large to be explained by these technical factors, indicating that there are genuine differences in Ca^2+^ influx magnitude between ribbons. Genuine ribbon-to-ribbon differences in response amplitude were also shown by feedback experiments in which the amplitude of ribbon-associated Ca^2+^ signals could be reversibly inhibited at one ribbon but not another in the same cone by activating feedback from postsynaptic HCs (e.g., Figure 8).

Salamander cone terminals do not exhibit Ca^2+^-induced Ca^2+^ release from intracellular stores (unlike rods: Krizaj et al., 2003; Cadetti et al., 2006) nor do these terminals possess mitochondria (Linton et al., 2010). Local differences in mitochondrial Ca^2+^ uptake or release of Ca^2+^ from endoplasmic reticulum stores are therefore not likely to be responsible for differences in voltage dependence or amplitude among ribbon-style active zones in salamander cones. Instead, these differences are more likely due to variability in Ca^2+^ influx through L-type Ca^2+^ channels clustered beneath individual ribbons (tom Dieck et al., 2005; Cadetti et al., 2006; Lv et al., 2012).

In addition to modulation of Ca^2+^ signals by HC feedback, there are many factors that could contribute to intrinsic ribbon-to-ribbon differences in Ca^2+^ influx. Differences in Ca^2+^ response amplitude at different ribbons could arise from differences in the number of Ca^2+^ channels at each ribbon. But in addition, Éltes et al. (2017) found significant differences in Ca^2+^ influx at different hippocampal terminals that exceeded differences in Ca^2+^ channel number, suggesting that differences in the function or subunit composition of Ca^2+^ channels also contribute to differences among active zones. The main Ca^2+^ channel subtype in rod and cone photoreceptors is Ca_V_1.4 but there is also evidence for Ca_V_1.3 channels (Xiao et al., 2007). Both of these channel types possess splice variants with altered voltage dependence (Bock et al., 2011; Tan et al., 2011; Haeseleer et al., 2016). Due to differences in the C-terminal domain, Ca_V_1.4 channels lack Ca^2+^-dependent inactivation (Wahl-Schott et al., 2006) and the inactivation properties of Ca_V_1.3 channels can vary among cells (Scharinger et al., 2015). It was suggested that interactions between Ca^2+^ channels and accessory Gipc3 proteins may contribute to observed differences in the voltage dependence of Ca^2+^ influx among ribbons in inner hair cells (Ohn et al., 2016). Similarly, differences in the voltage dependence and amplitude of Ca^2+^ signals at photoreceptor ribbons could potentially involve accessory proteins like CaBP4 (Haeseleer et al., 2004; Lee et al., 2014). Voltage dependence and conductance of photoreceptor Ca^2+^ channels can also be regulated by a host of neuromodulators including dopamine, cannabinoids, nitric oxide, somatostatin, fatty acids, and insulin (Stella and Thoreson, 2000; Vellani et al., 2000; Stella et al., 2001; Straiker and Sullivan, 2003; Kourennyi et al., 2004; Herrmann et al., 2011), the influence of which might vary among ribbons. Ca^2+^ channel voltage dependence and conductance are also regulated by local extracellular concentrations of Ca^2+^, H^+^, Zn^2+^, Cl^-^ or K^+^ ions (Wilkinson and Barnes, 1996; Cadetti et al., 2004; Cadetti and Thoreson, 2006; Babai and Thoreson, 2009). Ca^2+^ channels beneath ribbons are located at the apex of membrane invaginations that extend hundreds of nanometers into the cone terminal (Sterling and Matthews, 2005; tom Dieck et al., 2005; Lv et al., 2012). By isolating ribbon-associated Ca^2+^ channel clusters, this anatomical arrangement could promote local differences in the extracellular ionic microenvironment between invaginations. In the limited extracellular volume surrounding cone Ca^2+^ channels, small differences in the expression of a handful of ion channels, receptors, transporters, or binding partners could change the microenvironment enough to alter Ca^2+^ channel voltage dependence and channel conductance. Bipolar cells terminate in elaborately branched terminals possessing multiple presynaptic puncta (Euler et al., 2014). Ribbons at spatially distinct puncta in bipolar cells may also exhibit functional independence in their Ca^2+^ responses, especially given the relatively strong endogenous calcium buffers that are present in bipolar cells, equivalent to 1.2 mM BAPTA (Burrone et al., 2002).

In addition to differences in Ca^2+^ responses among ribbons, we found variability in the voltage dependence of I_Ca_ among cones, with a SD in V_50_ of 2.9 mV after accounting for trial-to-trial variability. The variance introduced by residual uncompensated R_ser_ was negligible. In isolated rods, voltage dependence of I_Ca_ shifted to more negative values over the first 3 min of whole cell recording as the pipette solution entered the cell (Corey et al., 1984). Time-dependent changes in voltage dependence may have contributed to the observed variability but we waited at least 3 min before making Ca^2+^ measurements and did not see consistent time-dependent changes. Differential effects of dopamine on I_Ca_ in small single vs. large single cones in salamander retina indicate that different cone subtypes can possess different Ca^2+^ channel types or key modulators (Stella and Thoreson, 2000). Immunohistochemical differences among medium and short wavelength-sensitive cones of tree shrew retina also suggest that Ca^2+^ channel expression may differ among cone subtypes (Morgans, 1999). Although these studies suggest that some of the differences in I_Ca_ voltage dependence we observed might arise from differences in Ca^2+^ channel subtypes or regulation among different classes of cones, we found no significant differences in I_Ca_ voltage dependence among the three cone subtypes examined in our studies.

Consistent with ultrastructural studies (Pierantoni and McCann, 1984; Ahnelt et al., 1990; Sterling and Matthews, 2005; Pang et al., 2008), we found that the size of ribbons measured with fluorescently-conjugated RIBEYE-binding peptides can vary appreciably. In inner hair cells, Ca^2+^ signal amplitude varied significantly among ribbons, with larger ribbons attaining higher depolarization-evoked Ca^2+^ levels (Frank et al., 2009; Ohn et al., 2016). We did not find a correlation between ribbon size and Ca^2+^ response amplitude, but did find that ribbon size was strongly correlated with the spatial extent of Ca^2+^ signals (Figure 4). This suggests that the average density of functional Ca^2+^ channels along the extent of a ribbon, which has been estimated at ~3 channels per vesicle in the readily releasable pool (Bartoletti et al., 2010; Thoreson et al., 2016), is similar for different-sized ribbons. While larger ribbons did not necessarily exhibit larger peak Ca^2+^ responses, they nevertheless experienced greater total Ca^2+^ influx because of the larger area of Ca^2+^ entry.

### Functional Consequences of Ribbon Variability

It has typically been thought that at the dark resting membrane potential, cone I_Ca_ is near the foot of its activation function (Barnes and Kelly, 2002). However, we found an average V_50_ for cone I_Ca_ of −38.1 mV, close to the average resting membrane potential for cones in darkness of −40 mV (Thoreson and Burkhardt, 1991). Because the I_Ca_/voltage activation curve is steepest at its V_50_, this arrangement is optimal for maximizing changes in release during small light-evoked changes in membrane voltage near darkness (Sterling and Laughlin, 2015). Such considerations also extend to local ribbon Ca^2+^ signals. At any given cone membrane potential, ribbons with V_50_ values near the membrane potential would exhibit the largest changes in synaptic output for a small change in voltage. Ribbon-to-ribbon differences in the voltage dependence of Ca^2+^ influx would therefore allow some ribbons to respond with greater sensitivity to light-evoked voltage changes under dim illumination, when the cone membrane potential is more depolarized, and other ribbons to respond with greater sensitivity in bright conditions when cones are more hyperpolarized. Individual bipolar and HCs receive inputs from many cone ribbons. Receiving an ensemble of inputs from ribbons with varying but overlapping sensitivities could improve the ability of bipolar and HCs to respond to contrasts over a wide range of light intensities.

Release of a vesicle at cone ribbon synapses requires the opening of an average of only 2–3 Ca^2+^ channels per vesicle (Bartoletti et al., 2011). A ribbon possessing more Ca^2+^ channels would be more likely to have an open channel and therefore release a vesicle at a given membrane voltage than a ribbon possessing fewer channels. This effectively makes ribbons with more Ca^2+^ channels more sensitive to small changes in membrane potential at hyperpolarized potentials, functioning much like a negative activation shift.

In the cochlea each postsynaptic spiral ganglion neuron contacts a single presynaptic inner hair cell ribbon, so variability among ribbons has been proposed to drive the diversity of spiking behaviors observed in spiral ganglion cells (Frank et al., 2009; Ohn et al., 2016). Unlike spiral ganglion neurons, bipolar and HCs in the retina receive inputs from many photoreceptor ribbons. It is therefore less likely that ribbon-to-ribbon differences in V_50_ and amplitude of Ca^2+^ responses would produce significant differences in the responses of second-order neurons (Burkhardt and Fahey, 1998; Burkhardt et al., 2006). To achieve such differences, the distribution a cell’s ribbon inputs would have to be skewed towards more positive or negative potentials. Postsynaptic differences in bipolar cell anatomy and physiology are more likely to be responsible for differences in their response properties. A number of postsynaptic factors have been identified as contributing to differences in response characteristics among bipolar cells, including differential expression of glutamate receptors and ion channels, dendritic anatomy (e.g., dendritic extent and position of postsynaptic contacts relative to the ribbon), and influence of lateral feedback from HCs to bipolar cells (DeVries et al., 2006; Szmajda and Devries, 2011; Thoreson and Mangel, 2012; Puller et al., 2013; Euler et al., 2014; Lindstrom et al., 2014). Although ribbon-to-ribbon variability seems unlikely to be a major factor in generating response differences among bipolar cells, cone-to-cone differences in both resting membrane voltage and the voltage dependence of whole cell I_Ca_ could produce differences among the responses of foveal midget bipolar cells in primate retina that receive inputs from only a single cone.

Endogenous Ca^2+^ buffering in cones is surprisingly weak, equivalent to only 50–100 μM EGTA (Van Hook and Thoreson, 2014). To improve the spatial resolution for detection of Ca^2+^ signals by confocal microscopy at individual ribbons, we used a higher concentration of EGTA (5 mM). Fast release of membrane-associated vesicles, as might occur during a rapid decrement in light, is regulated by Ca^2+^ within highly localized nanodomains <100 nm from Ca^2+^ channels even in the presence of weak exogenous buffering (Mercer et al., 2011; Van Hook and Thoreson, 2015). On the other hand, the slower sustained release that dominates in darkness is governed more strongly by the rate at which vesicles can be delivered to ribbons and ribbon-release sites (Jackman et al., 2009). The replenishment process that controls sustained release is itself regulated by Ca^2+^ acting at ribbon-associated sites located a few hundred nanometers above Ca^2+^ channels, and so sustained release is much more sensitive than fast release to Ca^2+^ buffering and global Ca^2+^ levels (Babai et al., 2010; Vaithianathan and Matthews, 2014; Van Hook et al., 2014; Van Hook and Thoreson, 2015). The impact of ribbon-to-ribbon differences in Ca^2+^ entry would therefore diminish during sustained release that occurs under conditions of dim illumination when Ca^2+^ levels are elevated similarly throughout the terminal.

HC activity is governed by glutamatergic inputs from presynaptic photoreceptors. Although there is controversy about the underlying mechanisms (Kramer and Davenport, 2015), there is an emerging consensus that changes in voltage dependence and amplitude of Ca^2+^ influx in cones caused by HC feedback involves extracellular proton levels within the synaptic cleft (Barnes and Bui, 1991; Hirasawa and Kaneko, 2003; Cadetti and Thoreson, 2006; Kemmler et al., 2014; Vroman et al., 2014; Warren et al., 2016a). Not every ribbon within a cone terminal was subject to feedback regulation from a single postsynaptic HC in our experiments. However, we found that 64% of the ribbons we analyzed in synaptically-connected cone-HC pairs were sensitive to feedback from a single postsynaptic HC. This was a higher percentage than predicted from previous work suggesting that a single HC in salamander retinal slices makes an average of 2.1 synaptic ribbon contacts per presynaptic cone (Bartoletti et al., 2010). Because salamander cones possess an average of 13 ribbons per cell (Bartoletti et al., 2010), we expected to see detectable feedback effects in fewer than 20% of ribbons. Furthermore, if only the few ribbons that directly contact an individual HC were influenced by its feedback, we would predict that the impact of feedback on whole cell I_Ca_—which is the summed currents from all the ribbons—would be less than 20% of the impact on Ca^2+^ responses at single ribbons. Instead, feedback-induced changes in the amplitude and voltage dependence of whole-cell I_Ca_ measured under similar experimental conditions in paired cone-HC recordings (Warren et al., 2016a) were only slightly smaller than changes observed at individual ribbons in the present study. The unexpectedly high prevalence of feedback effects suggests that the strong HC depolarization used to elicit negative feedback in these experiments may have spread through gap junctions to nearby coupled HCs, allowing feedback effects to become evident at ribbons that were not directly contacted by dendrites of the voltage-clamped HC. Much of the HC network is excised by slicing the retina into vertical sections, suggesting that this signal spread is likely to occur close to the recipient cone. The spread of voltage from one HC to another may contribute to the slower components of feedback seen in response to an abrupt change in HC membrane potential (Figure 8A; see also Warren et al., 2016b). These findings suggest that feedback effects from individual HCs are amplified by local interactions among HCs at photoreceptor synapses.

Our results show that Ca^2+^ signals at individual synaptic ribbons in cone photoreceptors can vary significantly from one another in both amplitude and voltage dependence. V_50_ values of ribbons within single cochlear inner hair cells varied with a SD of 3.2 mV (Frank et al., 2009; Ohn et al., 2016). Voltage dependence of inner hair cell ribbons also varied with subcellular location, with active zones on the modiolar side of inner hair cells activating at more positive voltages than pillar active zones. Thus, at least some of the differences among hair cell ribbons must arise from non-random processes (Ohn et al., 2016). Our results show that Ca^2+^ influx at individual cone ribbons can be rapidly and actively regulated by HC feedback in a non-random fashion. Intrinsic ribbon-to-ribbon differences in cones may also be actively regulated by cellular processes operating on a slow time scale. Alternatively, they may simply result from random differences in the expression and delivery of proteins to different parts of the cone. Regardless, ribbon-to-ribbon differences in Ca^2+^ signals expand the range of transformations available to cones for converting light-evoked voltage responses into synaptic release, and can thus expand the range of information provided to downstream neurons about changing light levels.

## Author Contributions

Both authors designed experiments, conducted experiments, analyzed data and wrote the article.

## Conflict of Interest Statement

The authors declare that the research was conducted in the absence of any commercial or financial relationships that could be construed as a potential conflict of interest.
