# Corrigendum: Synaptic Ribbon Active Zones in Cone Photoreceptors Operate Independently From One Another

**DOI:** 10.3389/fncel.2019.00541

**Published:** 2019-12-12

**Authors:** Justin J. Grassmeyer, Wallace B. Thoreson

**Affiliations:** ^1^Department of Pharmacology and Experimental Neuroscience, University of Nebraska Medical Center, Omaha, NE, United States; ^2^Truhlsen Eye Institute and Department of Ophthalmology and Visual Sciences, University of Nebraska Medical Center, Omaha, NE, United States

**Keywords:** ribbon synapse, retina, exocytosis, calcium imaging, cone photoreceptor, active zone

In the original article, there was a mistake in Figure 6D as published. The ordinate was not corrected for liquid junction potential. The corrected Figure 6 appears below.

**Figure 6:**
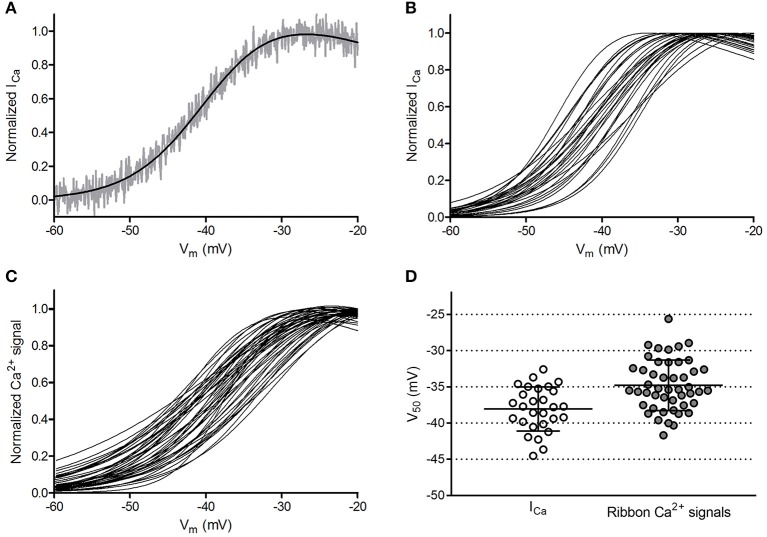
Ribbon-to-ribbon Ca^2+^ activation is more variable than cone-to-cone I_Ca_ activation. **(A)** I_Ca_ in one cone with R_ser_ completely compensated. I_Ca_ was normalized to its peak value and plotted against the cone holding potential during the voltage ramp protocol (gray trace). A Boltzmann function adjusted for driving force was fit to these data (black line, V_50_ = −39.3, slope factor = 4.88). Inward currents are plotted upward to compare more easily with Ca^2+^ signal measurements. For this illustration, currents were digitally corrected for the passive membrane resistance measured in the range from −85 mV to −70 mV. **(B)** Overlaid Boltzmann function fits to normalized I_Ca_ from the 28 cones in which ribbon Ca^2+^ signals in Panel **(C)** were measured. **(C)** Overlaid Boltzmann function fits to ribbon-associated Ca^2+^ signals of 47 ribbons in the 28 cones shown in Panel **(B)**. Ribbons were analyzed as illustrated in Figure 1. **(D)** Distribution of V_50_ values calculated from Boltzmann function fits to I_Ca_ (average = −38.1 ± 3.0 mV) and optical ribbon Ca^2+^ measurements made with OGB-5N (average = −34.8 ± 3.5 mV). Bars show the mean ± SD.

The authors apologize for this error and state that this does not change the scientific conclusions of the article in any way. The original article has been updated.

